# Supplementary material for: Folk arts-inspired twice-coagulated configuration-editable tough aerogels enabled by transformable gel precursors
Source: Nat Commun. 2023 Dec 19;14:8450. doi: 10.1038/s41467-023-44156-4 (PMC10730912; doi:10.1038/s41467-023-44156-4)
Supplement: Supplementary file 1 — Supplementary Information [file 41467_2023_44156_MOESM1_ESM.pdf]

Supplementary Information

Folk arts-inspired twice-coagulated configuration-editable tough aerogels enabled by transformable gel precursors

Lishan Li[1], Guandu Yang[1,2], Jing Lyu[1], Zhizhi Sheng[1], Fengguo Ma[2], and Xuetong Zhang[1,3,]*

[1]Suzhou Institute of Nano-Tech and Nano-Bionics, Chinese Academy of Sciences, Suzhou 215123, P. R. China
E-mail: xtzhang2013@sinano.ac.cn

[2]Key Laboratory of Rubber-Plastics (Ministry of Education), School of Polymer Science and Engineering, Qingdao University of Science and Technology, Qingdao 266042, P. R. China

[3]Division of Surgery & Interventional Science, University College London, London, NW3 2PF, UK

Supplementary Note 1. the impact of various factors on the TC process.

1) The ratio of ANF to PVA. To investigate the impact of the ANF to PVA ratio on the malleability of organogels, we examined the mechanical strength of AP organogels at various ANF/PVA ratios. The experimental design was: initially, different ratios of ANF and PVA (ANF/PVA=1/80, 1/40, 1/20, 1/10, 1/5, 1/2.5, and 1/1.25) were mixed. Supplementary Fig. 1 shows that gel formation occurred immediately after mixing when the ANF/PVA ratio was below 1/20 or above 1/1.25. At a ratio of 1/2.5, heterogeneous solutions with noticeable small gel particles were observed. The presence of gel monoliths and small gel particles indicated inadequate mixing between and PVA and ANF at these ratios, which were not suitable for TC strategy. After conducting more detailed experiments, we finally confirmed that elastic organogels could be formed within the range of ANF to PVA ratios from 1/15 to 5/1. When the ANF to PVA ratio exceeded 1:5 or fell below 1:15, insufficient mixing or excessive gelling occurred and the resulted organgels is too weak to be edited after freeze-thaw treatment (Supplementary Fig. 2). The possible reason is that excessive PVA content leads to protonation of ANF by hydrogen on the PVA, thereby promoting the formation of ANF gels. Conversely, an excessive amount of ANF induces gelation of PVA at room temperature due to the presence of excess alkali in ANF. Moreover, excessive ANF affects the crosslinking of PVA gels by wrapping or obstructing PVA molecular chains, consequently reducing the strength of organogels.

Subsequently, we conducted further investigations on the mechanical strength of organogels and hydrogels with an ANF/PVA ratio ranging from 1/15 to 1/5 (Supplementary Fig. 3). The strength of organogels increases gradually with the increase of ANF content. When the ANF/PVA ratio increases from 1/15, 1/10 to 1/5, the compression modulus increases from 5 kPa, 9 kPa to 14 kPa. The tensile elongation at break was significantly enhanced from 45% and 73% to 110%. This improvement can be attributed to the proportional increase in the number of cross-linking points of PVA induced by ANF. Similarly, the strength of hydrogels exhibited a positive correlation with ANF content, owing to the formation of a highly cross-linked hybrid network between ANF and PVA that effectively transfers applied stress within the hydrogel. The higher the content of ANF, the higher the crosslinking density and mechanical strength. Different from organic gels, the elongation at break of hydrogel decreases with the increase of ANF content. This is because in organogels with low crosslinking densities, the increasing crosslinking density can better resist stress and

reduce the risk of fracture. While in hydrogel with high crosslinking densities, as the crosslink density increased, the elastic PVA molecular chain length between crosslinkers decreased. According to rubber-like elasticity theory, the extensibility of elastomers is generally proportional to the number of monomer units between crosslinkers.[1,2] Therefore, the elongation at break of the AP hydrogel was reduced with the ANF content increase. [3]

2) Thermal history. To study the influence of the thermal history of ANF to PVA on malleable organogel, the mechanical strength of AP organogel by freezing-thawing with different times was tested respectively. The mechanical strength increases gradually with the increase of freezing-thawing times. (Supplementary Fig. 4.)

3) Ambient condition. The organogel is still malleable within 2 hours in ambient condition, however, it will lose its formative nature for a long time. Solvent drying is an inevitable process for most gels; however, in ambient conditions, here the absorption of humidity emerges as another influential factor. The weight of the organogel continues to increase in ambient conditions (25°C, 50% humidity), as demonstrated in Supplementary Fig. 5. This observation indicates the absorption of humidity from the air, ultimately leading to ANF protonation and rendering the organogel non-malleable. Consequently, it is necessary to store organogels under sealed conditions.

Supplementary equation 1. Calculation of PVA/ANF.

As TGA shown in figure 1e, the mass decomposed at 250 °C-500 °C of pure PVA m (PVA, 250-500 °C) accounts for 90%, so the total mass of PVA is m (PVA, 250-500 °C)/90%. The mass decomposed at 520-620 °C of pure ANF m (ANF, 520-620 °C) accounts for 50%, so the total mass of ANF is m (ANF, 520-620 °C)/50%. The TGA curve of AP at 250 °C to 500 °C is attributed to the decomposition of PVA, and the decomposition mass is 75% m (AP), so the total mass of PVA in AP is 75% m (AP)/90%. Similarly, the decomposition curve of AP at 520-620 °C is attributed to the decomposition of ANF (PVA no longer decomposes at 520-620 °C), and the decomposition mass is 8.4% m(AP), so the total mass of ANF in AP is calculated as 8.4% m (AP)/50%. So the ratio of PVA/ANF is [75% m(AP)/90%]/[8.4% m(AP)/50%],

namely (75% / 90%)/(8.4% / 50%). It can be expressed by the Supplementary equation:

$$\frac{m(PVA)}{m(ANF)} = \frac{\frac{m(AP,\ 250-500)}{90\%}}{\frac{m(AP,520-620\,)}{50\%}} = \frac{75\%\, m(AP)/90\%}{8.4\%\, m(AP)/50\%} = 5/1 \qquad 1)$$

Supplementary Figures

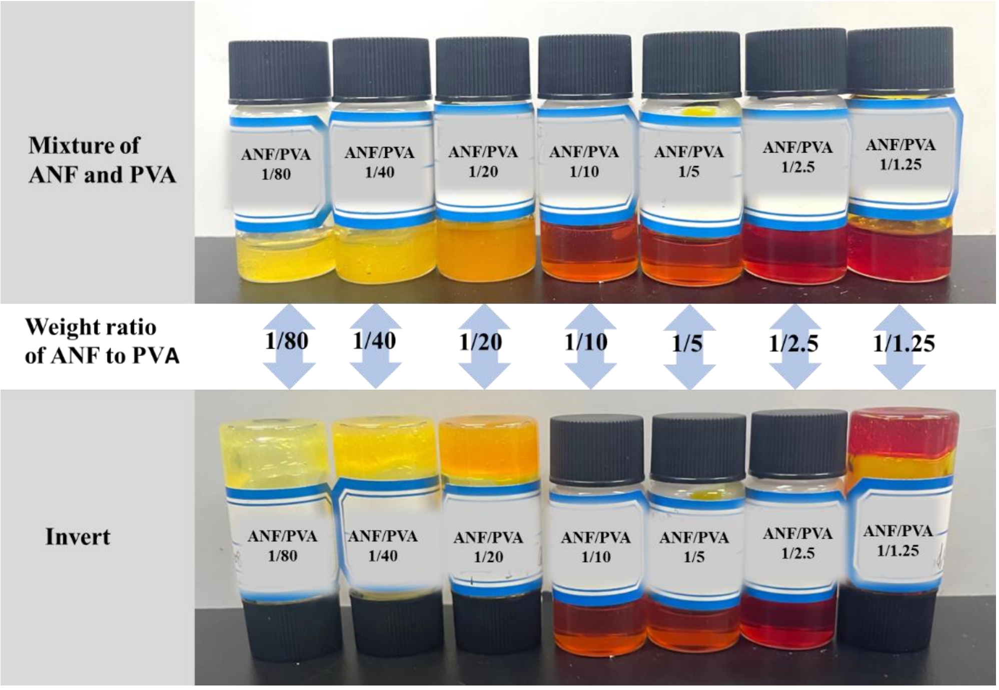

Supplementary Fig. 1. Mixtures of ANF and PVA with different ratios. The ratios of ANF to PVA were 1/80, 1/40, 1/20, 1/10, 1/5, 1/2.5, 1/1.25 respectively.

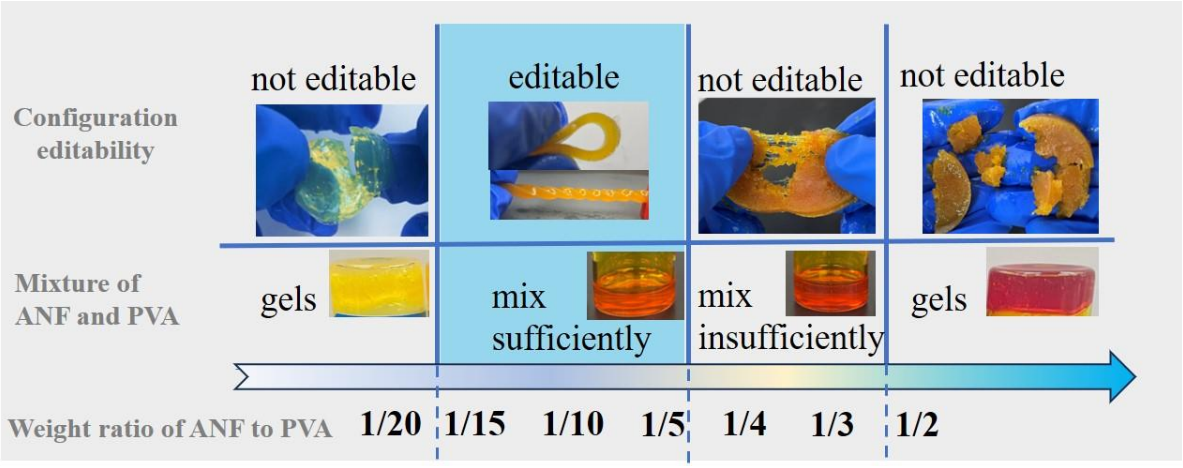

Supplementary Fig. 2. Mixtures of ANF and PVA and the configuration editability. The appropriate ratios of ANF to PVA for mixtures with configuration editability ranged

from 1/15 to 1/5.

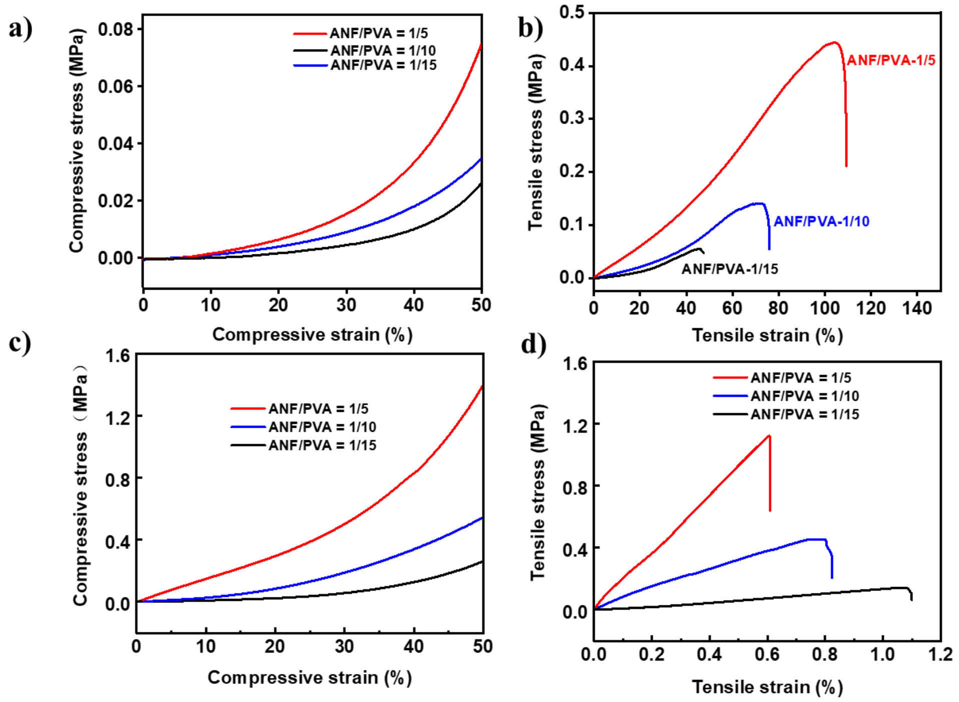

Supplementary Fig. 3. Compressive and tensile stress-strain curves of organogels and hydrogels. a Compressive curves. **b** Tensile stress-strain of AP organogels with different ratio of ANF/PVA. **c** Compressive curves. **d** Tensile stress-strain of AP hydrogels with different ratio of ANF/PVA.

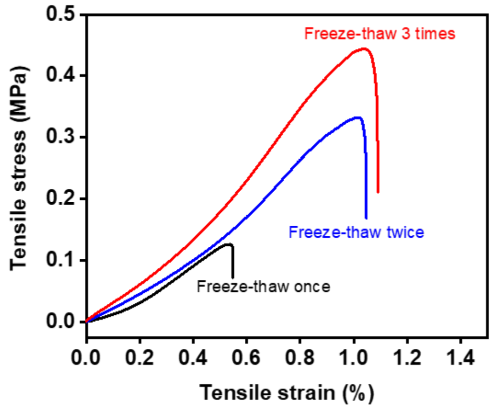

Supplementary Fig. 4. Tensile stress-strain curves of AP organogels with different freeze-thaw cycles . The ratio of ANF/PVA is 1/5, and the freeze-thaw cycles were once, twice and three times.

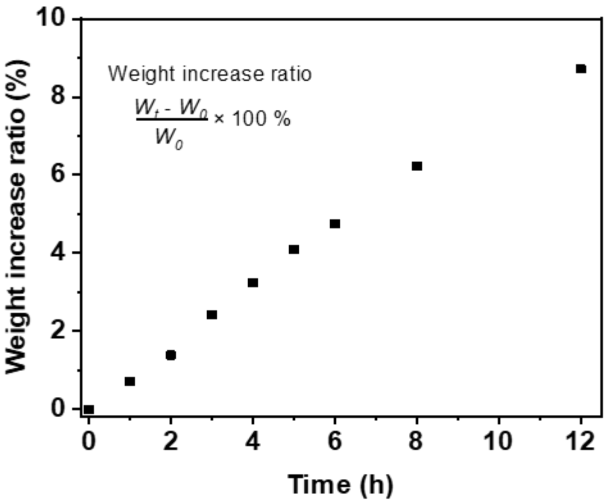

Supplementary Fig. 5. The weight variation of AP organogels exposed in ambient condition (25 °C, 50% humidity). W_o is the initial weight of sample before exposing in ambient condition, and W_t is the real-time weight of sample after exposing in ambient condition.

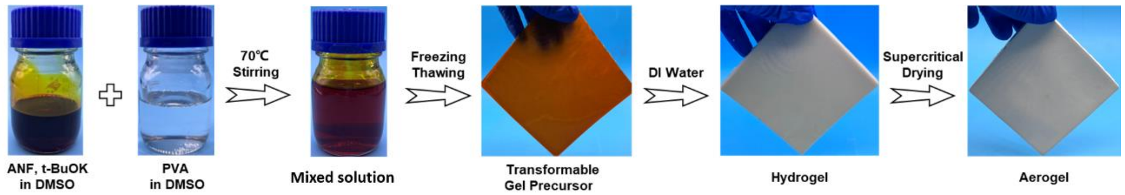

Supplementary Fig. 6. Preparation process of AP organic gel, hydrogel, and aerogel. The specific experimental steps are described in the experimental section. Kevlar 1000D was dissolved in KOH/t-BuOK DMSO under magnetic stirring at 25 °C for 7 days to obtain a dark red, viscous solution of Aramid nanofibers (KNFs) (2.0 wt%).

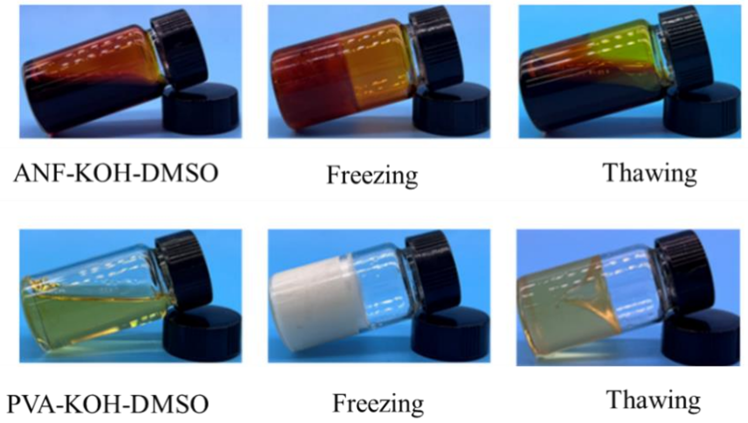

Supplementary Fig. 7. ANF-KOH-DMSO and PVA-KOH-DMSO undergoing freeze-thaw treatment. ANF-KOH-DMSO means ANF dissolved in KOH DMSO solution, and PVA-KOH-DMSO means PVA dissolved in KOH DMSO solution.

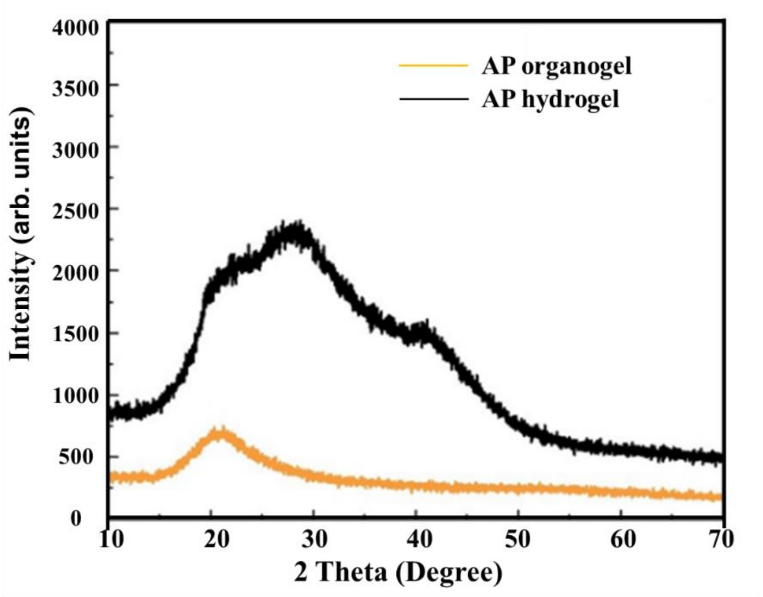

Supplementary Fig. 8. XRD patterns of AP organogel and hydrogel. Compared with the weak diffraction near 20° in organogel, attributing to the crystallization in Aramid nanofibers or PVA crystallization, the stronger diffraction and new forming peak near 40 degrees indicates the further crystallization of PVA chains in hydrogel.

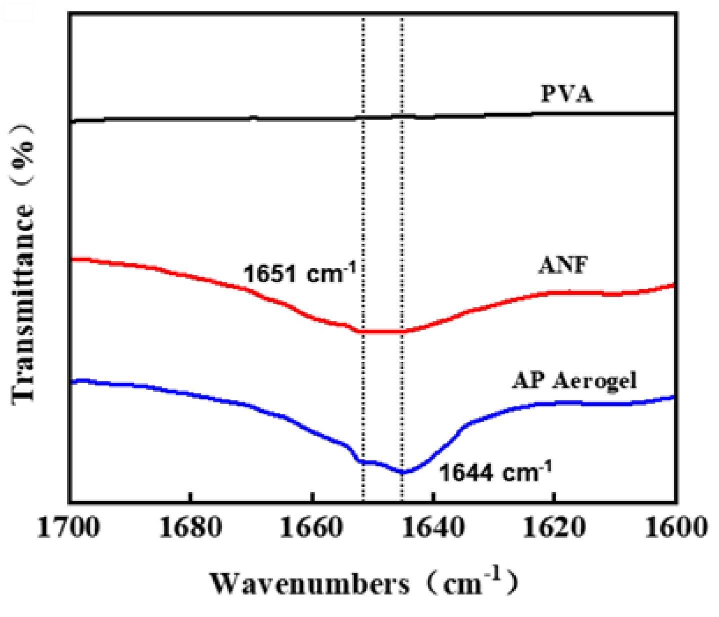

Supplementary Fig. 9. FTIR spectra showing the peak positions associated with the aramid C=O stretching vibration. The C=O stretching vibration peak at 1651 cm^{-1} of AP partly moves to 1644 cm^{-1}, indicating the existence of hydrogen bonds among AP.

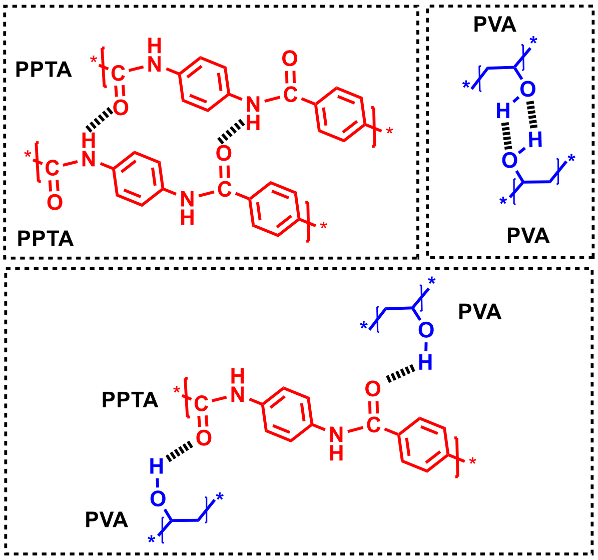

Supplementary Fig. 10. Illustration of molecular interaction among AP in the process of secondary coagulation. Multiple interactions of PVA-PVA, ANF-ANF and ANF-PVA coexist to form a dense cross-linked network.

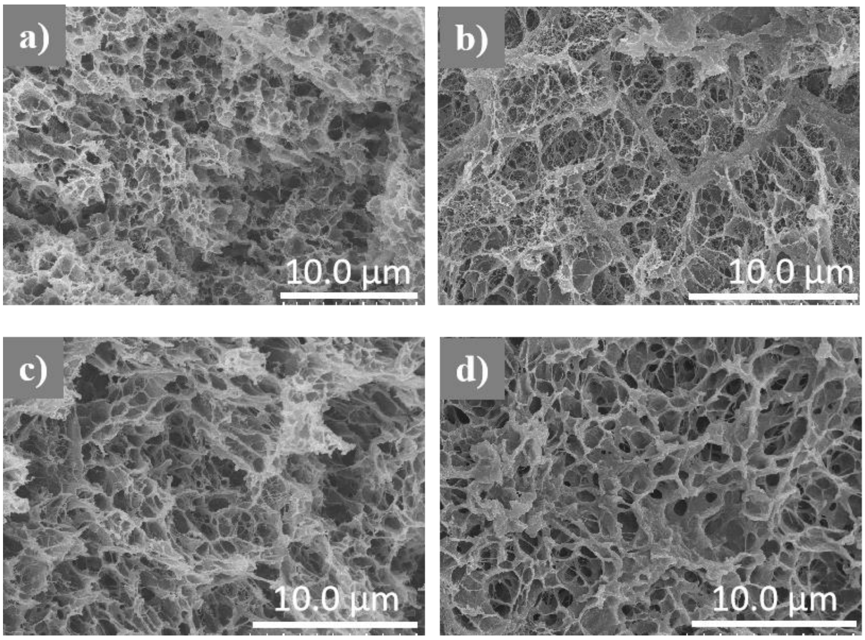

Supplementary Fig. 11. SEM images of AP aerogels. a AP-17, **b** AP-19, **c** AP-25, **d** AP-27 aerogel undergoing three freeze-thaw cycles. All the cellular network skeletons consist of Aramid nanofibers surrounded by PVA wall.

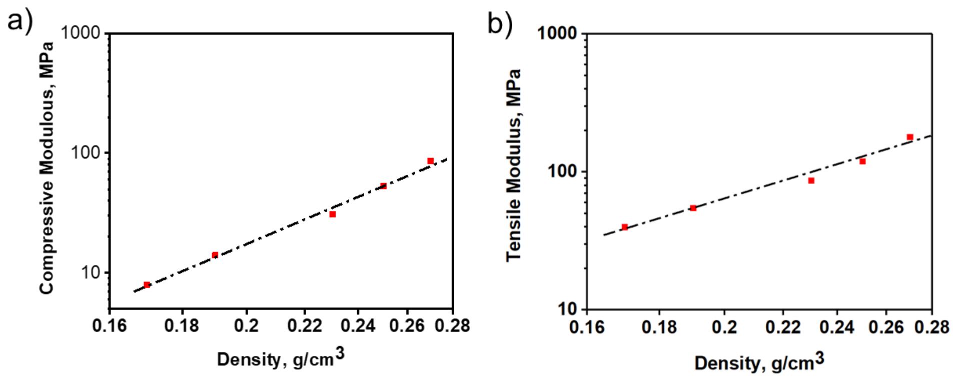

Supplementary Fig. 12. The log−log plot of modulus vs density of AP aerogels. **a** log−log plot of compressive modulus vs density (log standard deviation = 0.25, R^2 = 0.99). **b** log−log plot of tensile modulus vs density (log standard deviation = 0.27, R^2 = 0.97).

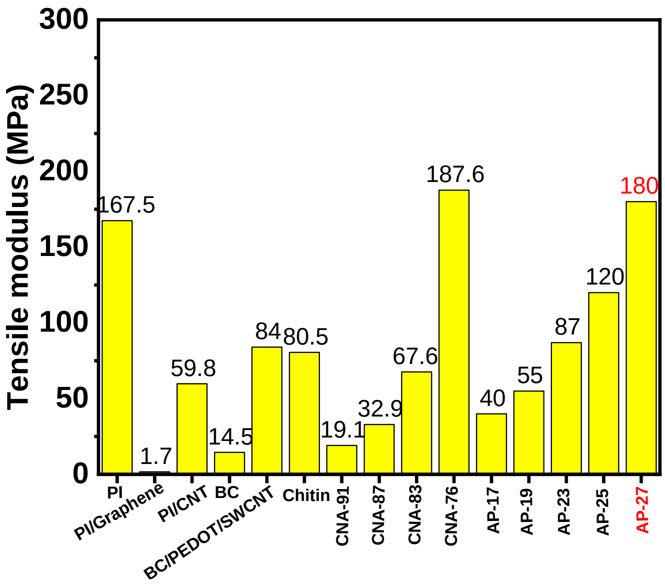

Supplementary Fig. 13. The comparison of tensile modulus between high-strength polymeric polymers. The tensile modulus of AP-27 aerogel reaches 180 MPa.

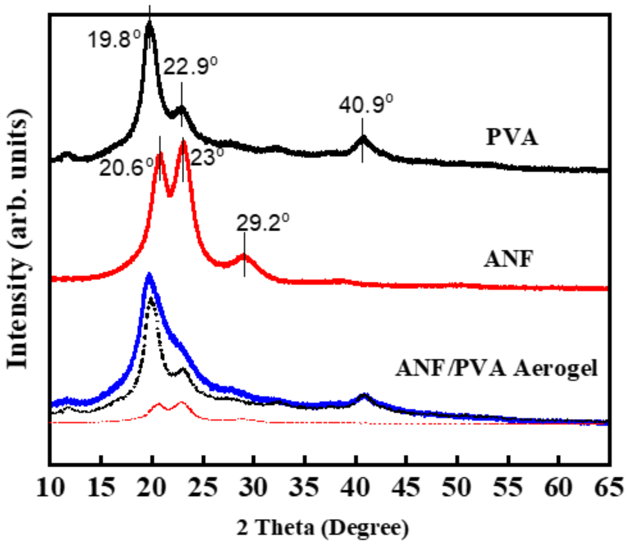

Supplementary Fig. 14. XRD patterns of PVA, ANF, AP aerogel. The diffraction pattern of the aerogel consists of major PVA and minor ANF diffraction peaks, which indicates the presence of PVA and ANF crystals in the aerogel.

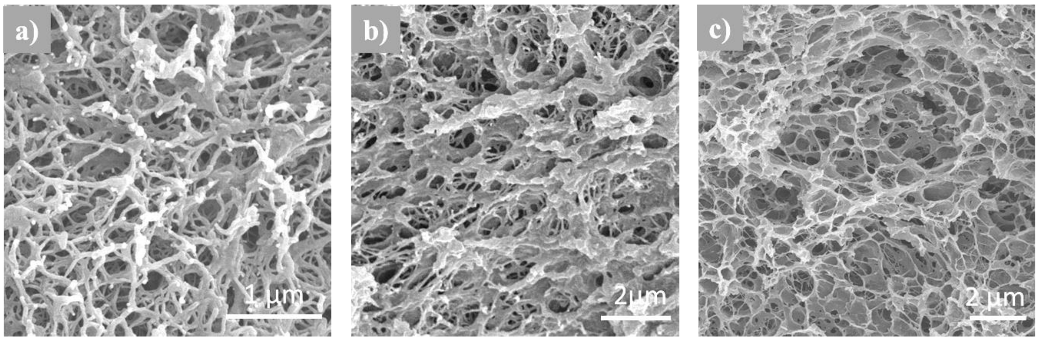

Supplementary Fig. 15. SEM images of AP-23 aerogels from organogels undergoing different freeze-thaw cycles. **a** zero freeze-thaw cycle (AP-FT-0). **b** one freeze-thaw cycle (AP-FT-1). **c** three freeze-thaw cycles (AP-FT-3).

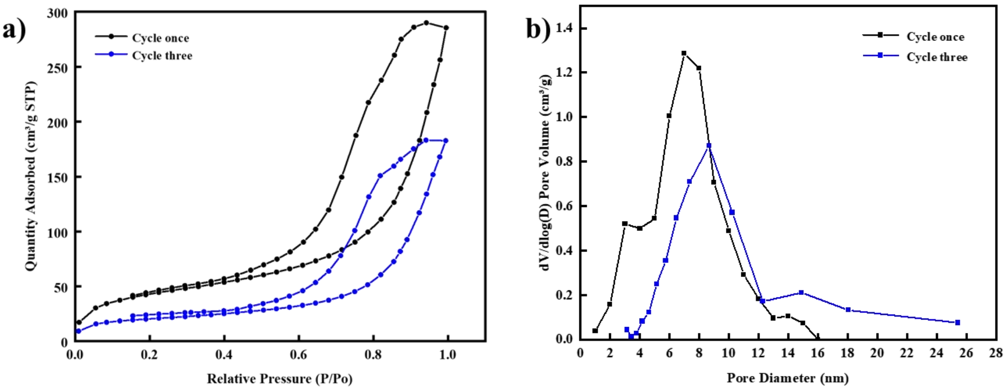

c)

AP aerogel, one freeze-thaw cycle	AP aerogel, three freeze-thaw cycles
BET Surface Area: 153.6503 m²/g	BET Surface Area: 70.0800 m²/g
Pore Volume: 0.451956 cm³/g	Pore Volume: 0.284824 cm³/g
Pore Size: 11.49648 nm	Pore Size: 16.12668 nm

Supplementary Fig. 16. Mesoporous analysis of AP aerogels. **a** Nitrogen adsorption-desorption isotherm. **b** pore size distribution. **c** data comparison of AP-23 aerogel treated by one and three freeze-thaw cycles. The decreased BET surface area and increased pore size after three freeze-thaw cycles indicates the aggregation of nanofibers.

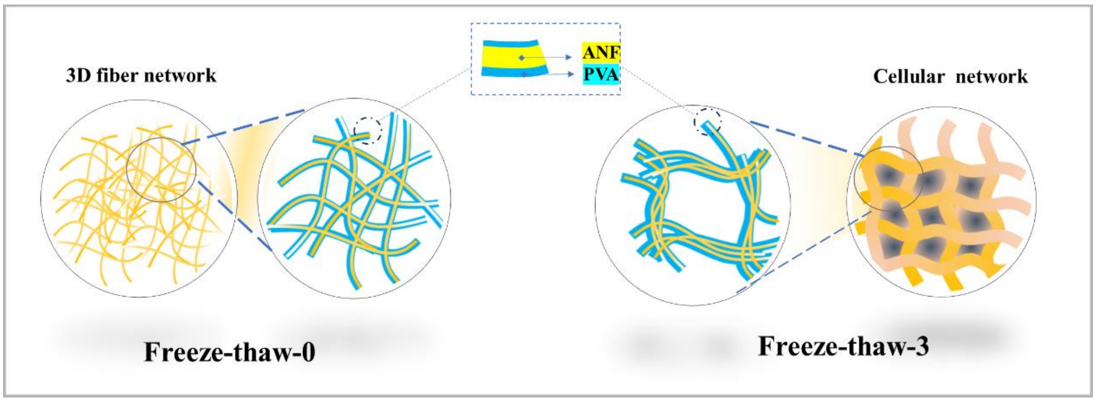

Supplementary Fig. 17. Schematic diagram of the difference between 3D fiber network and cellular network.

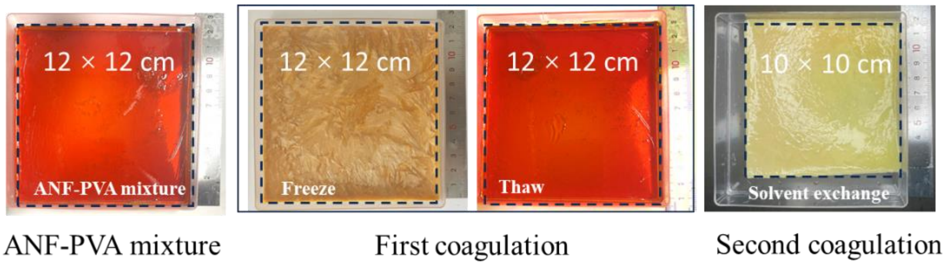

Supplementary Fig. 18. Volume shrinkage during tiwce coagulation process. In

first coagulation process, no significant changes in configuration were observed. During solvent exchange processes in the second coagulation step, a volume shrinkage of approximately 40% occurred.

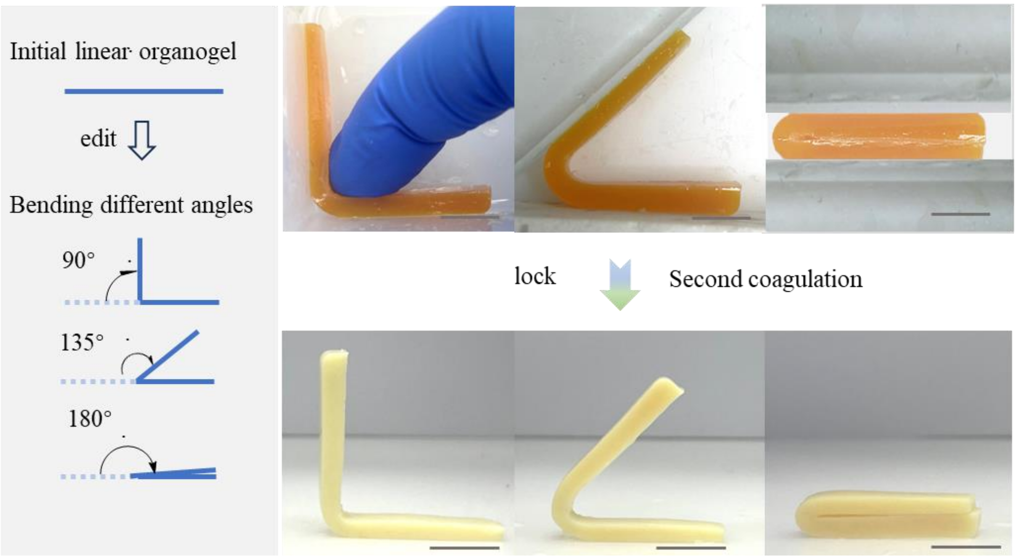

Supplementary Fig. 19. Shape retention of the bending gels. The bending angle of 90°, 135°, 180° of the bended rectangular spline remains unchanged after secondary coagulation. Scale bar, 1cm.

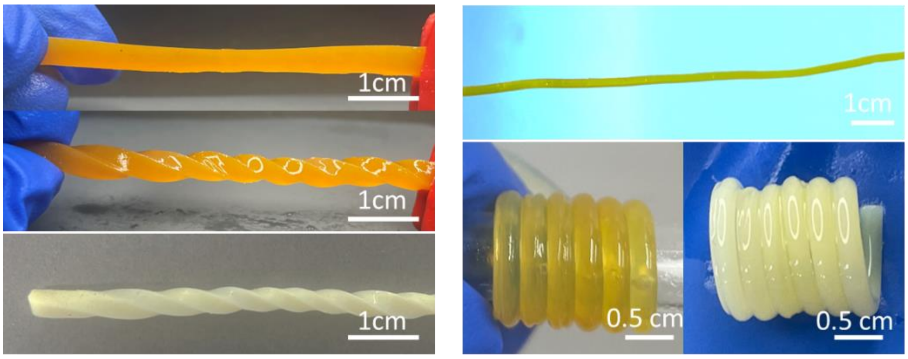

Supplementary Fig. 20. Shape retention of the twisting and twining gels. The organogels with twisting and twining editing could also keep the oringinal configurations after secondary coagulation.

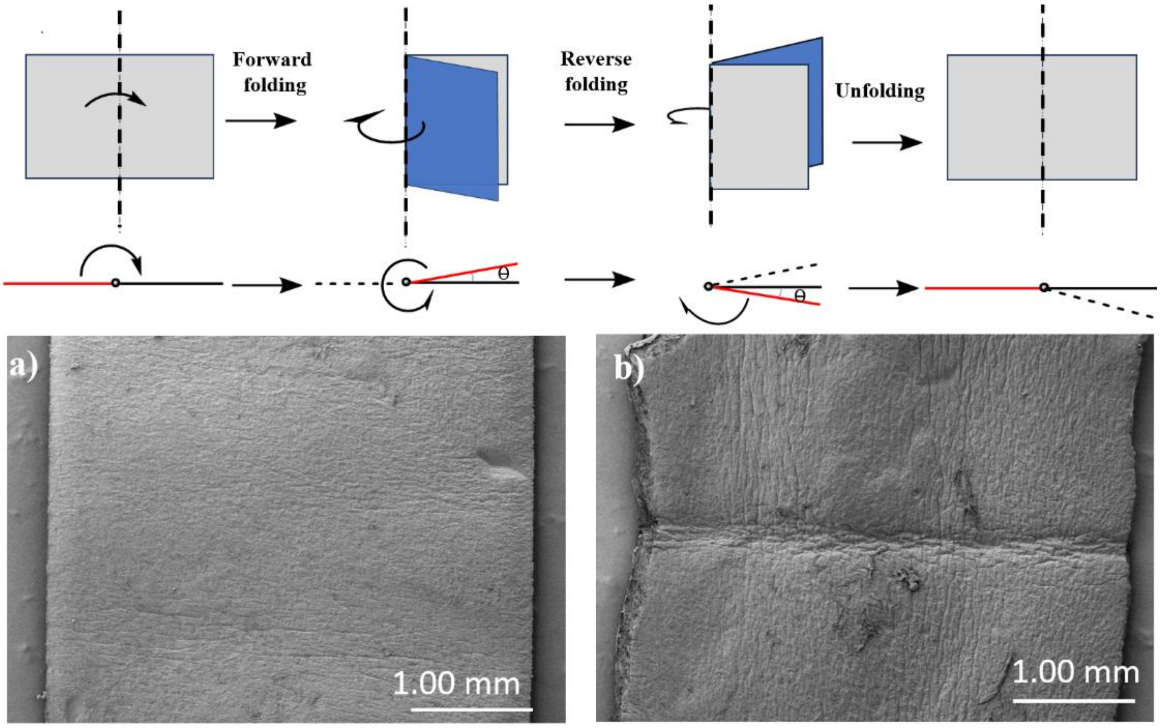

Supplementary Fig. 21. SEM images of AP-23 aerogels at folding location. a The origami by twice-coagulated strategy. **b** Origami by folding aerogel film directly. No significant cracks were observed in Supplementary Fig. 21a, while the aerogel film in Supplementary Fig. 21b would crease after folding.

Origami by TC strategy: Organogel film - Forward folding (the inner corner of the folded area is zero) - Reverse folding (the inner corner of the folded area is zero) - Unfolding (for SEM observation) - Dry.

Origami by folding Directly: Aerogel film - Forward folding (the inner corner of the folded area is zero) - Reverse folding (the inner corner of the folded area is zero) - Unfolding (for SEM observation)

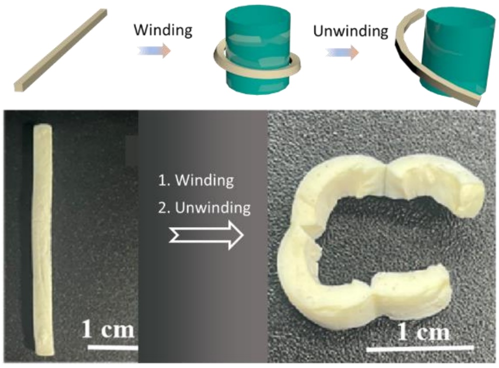

Supplementary Fig. 22. Aerogel rod and the broken aerogel after directly editing. Different from flexible organic rods, irreversible structural damage occurs after stretching, compression, bending, and twisting aerogel rods. The cracking would occur

if directly editing aerogel such as stretching, compression, bending, and twisting aerogel. For example, in Supplementary Fig. 22, when winding the rectangular spline with 2 mm×2 mm cross section along a cylinder with 8mm diameter and unwinding outward, the aerogel spline is cracked.

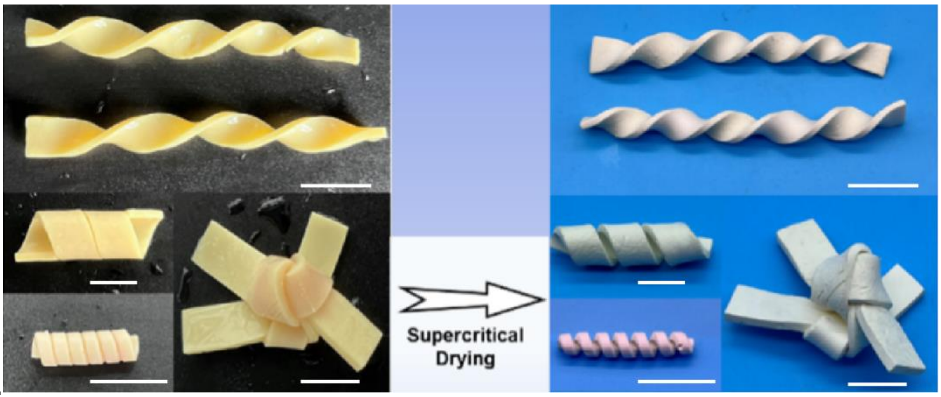

Supplementary Fig. 23. Hydrogels and aerogels with specific configurations. Configurations were edited by twisting, wending and knotting respectively. Scale bar: 1 cm.

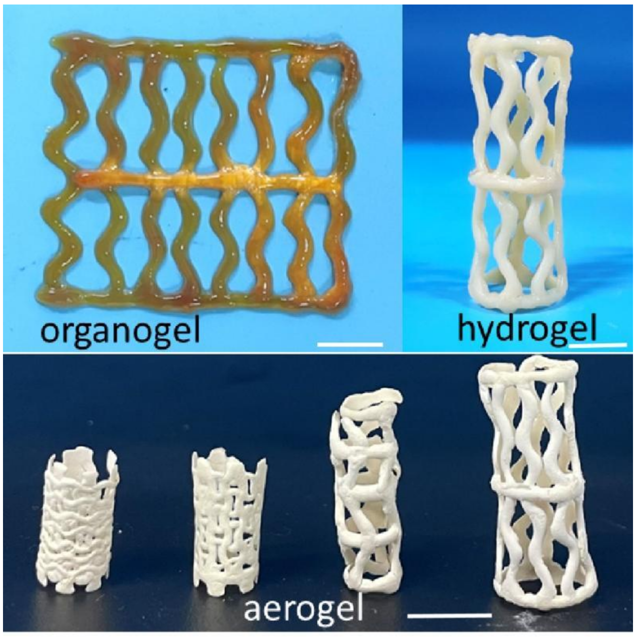

Supplementary Fig. 24. Preparation of three-dimensional (3D) hollow aerogels. First, the two-dimensional pattern is drawn with ANF and PVA mixed solution. After freezing and thawing, the obtained two-dimensional organic gel is deformed, such as bending, and then the shape is fixed by secondary solidification in deionized water. Then the 3D hollow aerogels are obtained by supercritical drying. Scale bar: 1 cm.

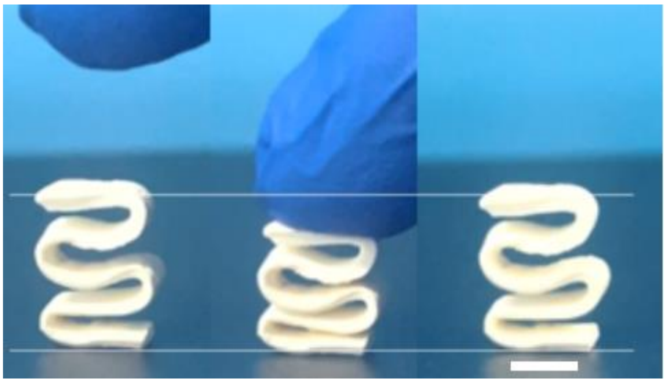

Supplementary Fig. 25. Compressive resilience of wavy aerogel. The aerogel spring is compressed with the finger to 25% strain, then the spring deformation is restored after releasing finger (scale bar: 1 cm).

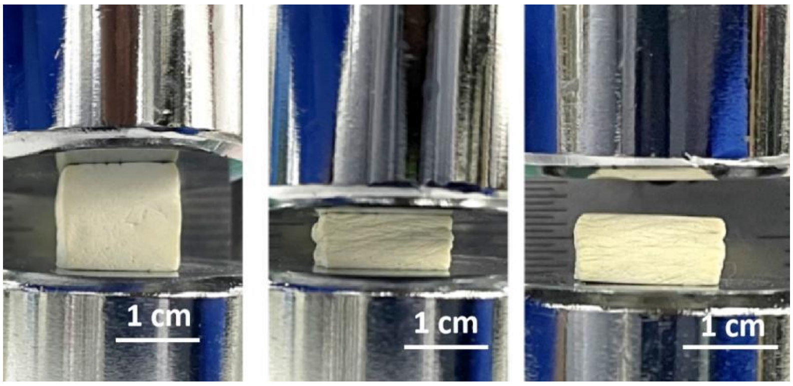

Supplementary Fig. 26. Photographs of AP aerogel monolith before and after compression. The aerogel monolith is compressed to 25% strain, then deformation could not be restored after releasing compression.

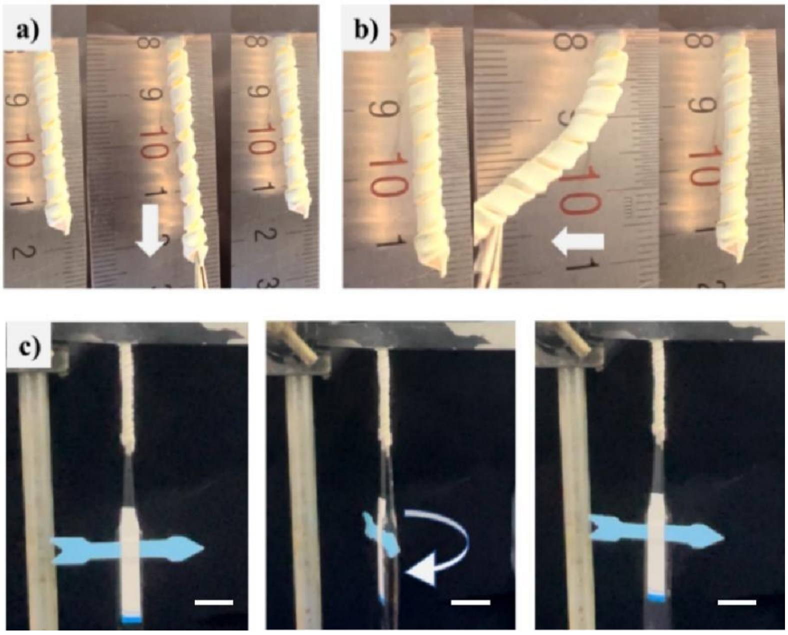

Supplementary Fig. 27. Deformation resilience of aerogel springs. **a** After stretching 25% tensile strain. **b** Bending 45 degrees. **c** Twisting 90 degrees, the

deformation of the aerogel spring can be restored. Scale bar: 1 cm.

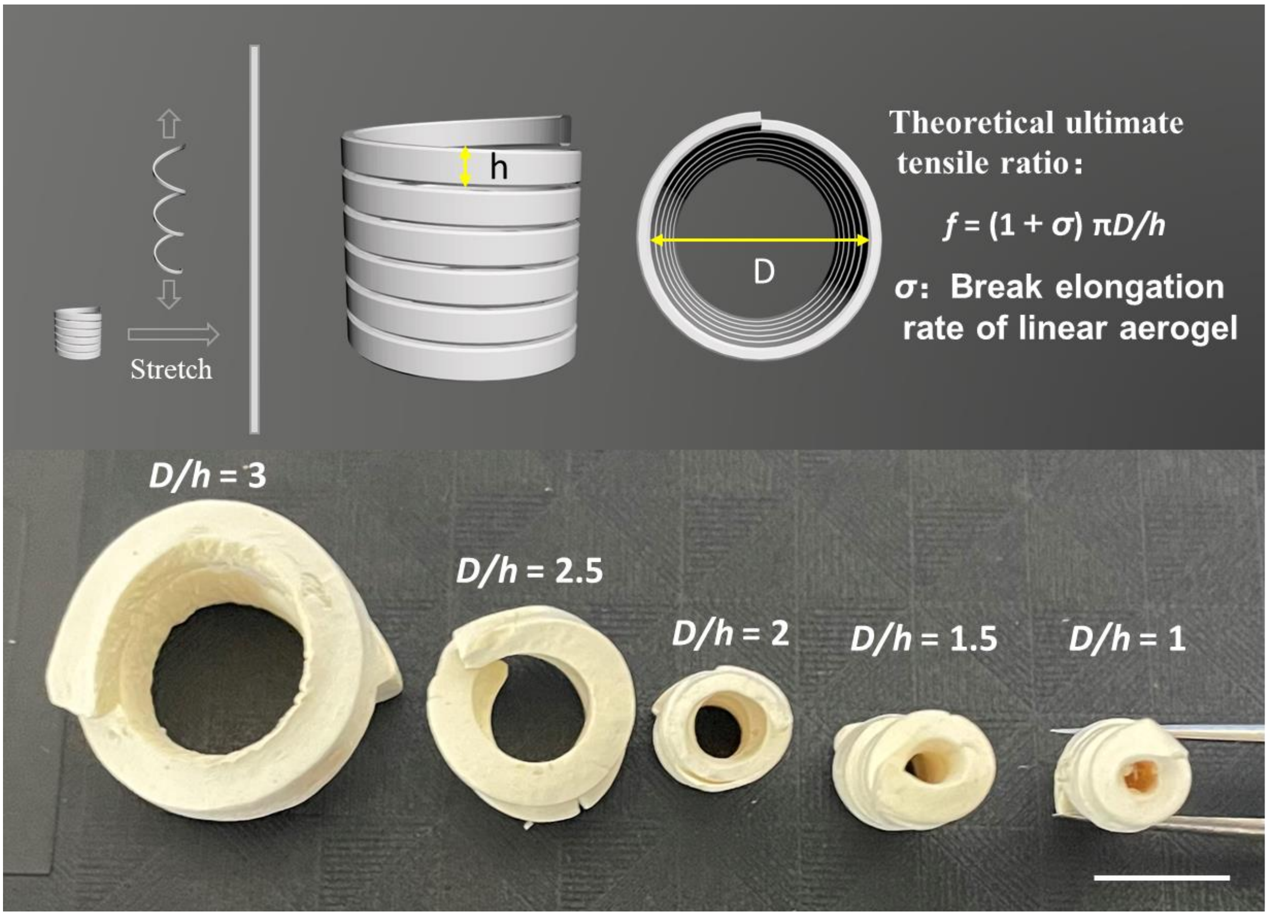

Supplementary Fig. 28. Schematic diagram and aerogel springs. Schematic diagram of the ultimate tensile process and estimated theoretical ultimate tensile ratio of aerogel spring and the prepared aerogel springs with different ratio of diameter (D) to height (h). Theoretical ultimate tensile ratio: $f = (1 + \sigma)\ \pi D/h$. where σ is break elongation rate of linear aerogel, D is the diameter, and h is the height of the pitch. The elongation is contributed by two parts, one is the elongation of the spring being straightened $\pi D/h$, and the other is the elongation at break of the straight aerogel $\sigma\pi D/h$. Scale bar: 1 cm.

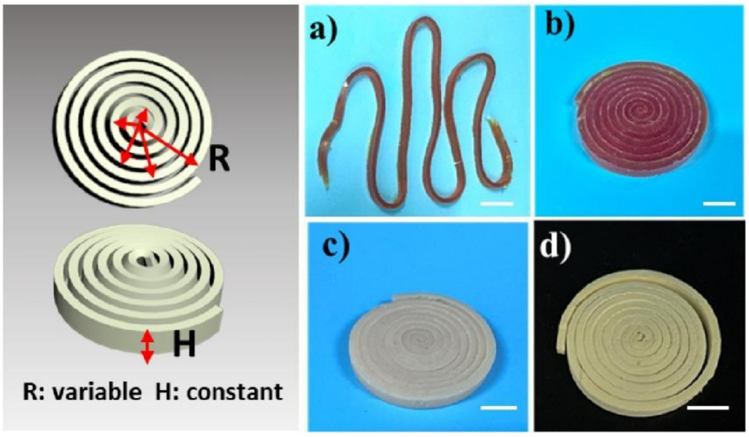

Supplementary Fig. 29. Parameters and preparation process of coil aerogel. The radius (R) is variable and the height (H) is constant. The coil aerogel could be obtained by **a, b** rotating AP organogel splines concentrically, **c** locking shape in deionized water, exchanging solvent in ethanol and **d** drying in supercritical CO_2. Scale bar: 1 cm

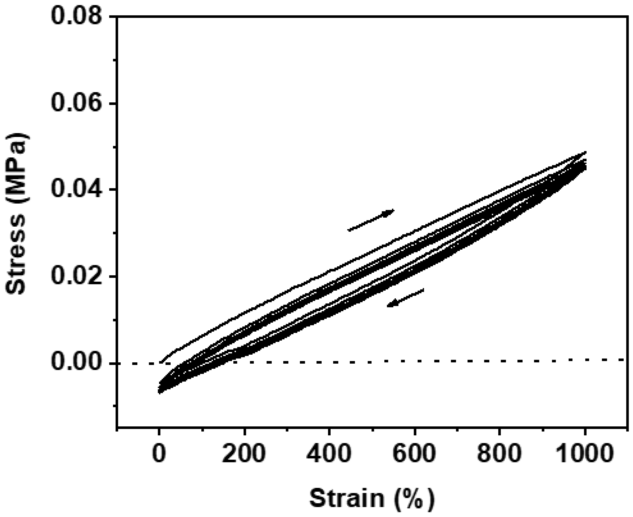

Supplementary Fig. 30. Complete tensile cyclic curves of coil aerogels with 1000% strain. The details of the measurement: we define the “zero” as 0% strain. The stretching procedure is as follows: Each cycle starts with an initial 0% tensile strain, then stretches 20 mm /min to a preset tensile strain value (e.g., 1000%), then 20 mm /min returns to 0% tensile strain, and then repeat the process for the next cycle. Under large tensile strain, the deformation of aerogel includes elastic deformation of spring and intrinsic plastic deformation of ANF aerogel. Elastic deformation predominates as the primary mode of deformation, while plastic deformation acts as secondary one. Consequently, the tensile cycle curve exhibits near-linearity due to the dominant elastic spring-like behavior, whereas the hysteresis curve fails to return to zero owing to the presence of AP aeogel's intrinsic plasticity.

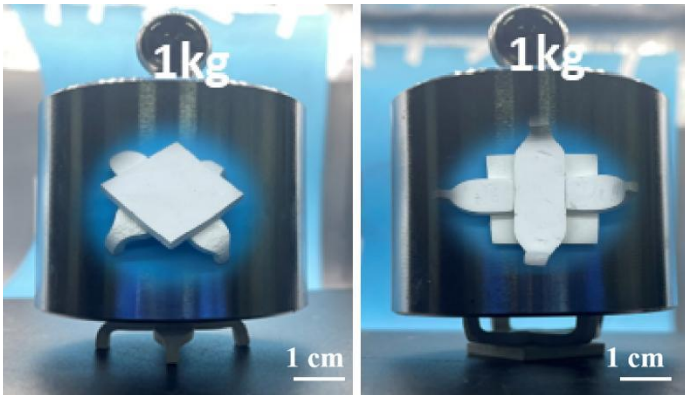

Supplementary Fig. 31. Tough aerogel scaffold bearing support. Aerogel table with four feet could support 1kg load easily without obvious deformation.

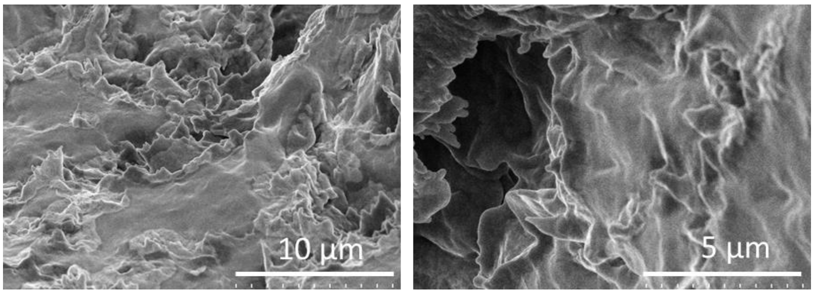

Supplementary Fig. 32. SEM images of Paraffin@AP. The pores of the aerogel were filled with paraffin.

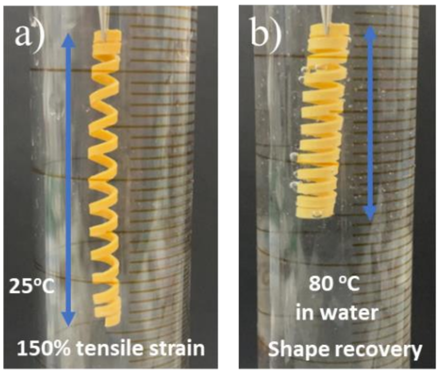

Supplementary Fig. 33. Shape memory of Paraffin@aerogel spring in hot water
a Paraffin@aerogel stretched and locked with 150% tensile strain. **b** Rapid shape recovery in 80 °C hot water.

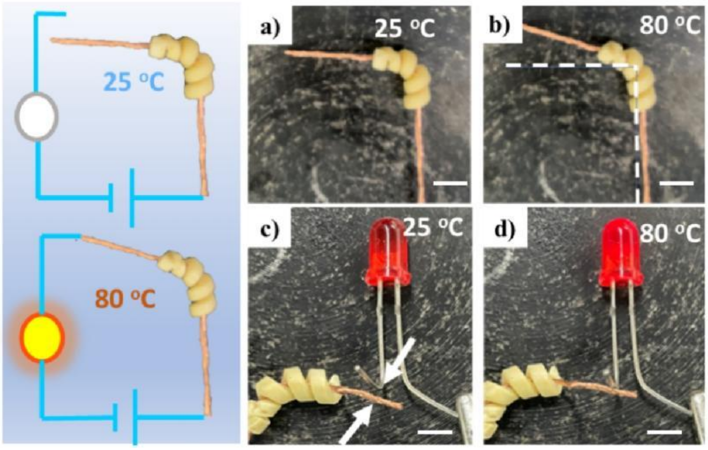

Supplementary Fig. 34. Paraffin@aerogel using as actuator for electronic switch.
a, b The uncurling process of bended Paraffin@aerogel spring covering copper wire.
c, d Actuator for electronic switch by connecting circuit at 80 °C via shape memory. Scale bar: 1 cm.

Supplementary Tables

Supplementary Table 1. Toughness, specific tensile modulus and other parameters of AP aerogels with different densities, as compared with other high-strength polymeric aerogels. [4-11]

	Density ($g\ cm^{-3}$)	Tensile modulus (MPa)	Specific tensile modulus ($MPa\ cm^3\ g^{-1}$)	Toughness ($kJ\ m^{-3}$)	Ref.
Crosslinked Polyimide (PI)	0.3	167.5	558.3	362.3	4
PI/Graphene	0.01	1.7	173.8	9	5
PI/CNT	0.4	59.8	149.5	152.8	6
Bacterial Cellulose (BC)	0.32	14.5	45.2	207.4	7
BC/PEDOT/ SWCNT	0.35	84	240	22.7	8
Chitin	0.17	80.5	473.5	480.7	9
Chitosan	/	/	550.6	147.6	10
CNA-91	0.14	19.1	136.4	212	11
CNA-87	0.16	32.9	205.6	406	11
CNA-83	0.21	67.6	321.9	523	11
CNA-76	0.30	187.6	625.3	1050.6	11
AP-17	0.17	40	235	654	this work
AP-19	0. 19	55	289	906	this work
AP-23	0.23	87	378	1344	this work
AP-25	0.25	120	480	1543	this work
AP-27	0.27	180	666	2093	this work

Note: most of the data refer to the literature 11.

Supplementary References:

1 Zhang, Z. *et al.* Surpassing the stiffness-extensibility trade-off of elastomers via mastering the hydrogen-bonding clusters. *Matter* **5**, 237-252 (2022).

2 Zhao, X. *et al.* Soft Materials by Design: Unconventional Polymer Networks Give Extreme Properties. *Chem. Rev.* **121**, 4309-4372 (2021).

3 Xing, H. Z. *et al.* Strong, tough, fatigue-resistant and 3D-printable hydrogel composites reinforced by aramid nanofibers. *Mater. Today* **68**, 84-95 (2023).

4 Guo, H. *et al.* Tailoring Properties of Cross-Linked Polyimide Aerogels for Better Moisture Resistance, Flexibility, and Strength. *ACS Appl. Mater. Interfaces* **5**, 225-225 (2013).

5 Qin, Y. *et al.* Lightweight, Superelastic, and Mechanically Flexible Graphene/Polyimide Nanocomposite Foam for Strain Sensor Application. *ACS Nano* **9**, 8933-8941 (2015).

6 Liu, P., Tran, T. Q., Fan, Z. & Duong, H. M. Formation mechanisms and morphological effects on multi-properties of carbon nanotube fibers and their polyimide aerogel-coated composites. *Compos. Sci. Technol.* **117**, 114-120 (2015).
7 Wan, J., Zhang, J., Yu, J. & Zhang, J. Cellulose Aerogel Membranes with a Tunable Nanoporous Network as a Matrix of Gel Polymer Electrolytes for Safer Lithium-Ion Batteries. *ACS Appl. Mater. Interfaces* **9**, 24591-24599 (2017).
8 Jia, F. *et al.* High Thermoelectric and Flexible PEDOT/SWCNT/BC Nanoporous Films Derived from Aerogels. *ACS Sustainable Chem. Eng.* **7**, 12591-12600 (2019).
9 Ding, B. *et al.* Light weight, mechanically strong and biocompatible alpha-chitin aerogels from different aqueous alkali hydroxide/urea solutions. *Sci. China. Chem.* **59**, 1405-1414 (2016).
10 Gong, Y. *et al.* Synthesis and Characterization of Graphene Oxide/Chitosan Composite Aerogels with High Mechanical Performance. *Polymers* **11**, 777 (2019).
11 He, H. *et al.* Ultrastrong and multifunctional aerogels with hyperconnective network of composite polymeric nanofibers. *Nat. Commun.* **13**, 4242 (2022).
