# Supplementary material for: Folk arts-inspired twice-coagulated configuration-editable tough aerogels enabled by transformable gel precursors
Source: Nat Commun. 2023 Dec 19;14:8450. doi: 10.1038/s41467-023-44156-4 (PMC10730912; doi:10.1038/s41467-023-44156-4)
Supplement: Supplementary file 3 — Description of additional supplementary files [file 41467_2023_44156_MOESM3_ESM.pdf]

<u>Description of additional supplementary files</u>

Supplementary Movie 1: Shape memory of Paraffin@aerogel spring after undergoing stretching, compressing, twisting and bending.

Supplementary Movie 2: Shape memory of Paraffin@aerogel spring in hot water.
